# Epigenetics: The New Frontier in the Landscape of Asthma

**DOI:** 10.1155/2016/4638949

**Published:** 2016-05-11

**Authors:** Bharti Chogtu, Dipanjan Bhattacharjee, Rahul Magazine

**Affiliations:** ^1^Department of Pharmacology, Kasturba Medical College, Manipal University, Karnataka 576104, India; ^2^Department of Pulmonary Medicine, Kasturba Medical College, Manipal University, Karnataka 576104, India

## Abstract

Over the years, on a global scale, asthma has continued to remain one of the leading causes of morbidity, irrespective of age, sex, or social bearings. This is despite the prevalence of varied therapeutic options to counter the pathogenesis of asthma. Asthma, as a disease per se, is a very complex one. Scientists all over the world have been trying to obtain a lucid understanding of the machinations behind asthma. This has led to many theories and conjectures. However, none of the scientific disciplines have been able to provide the missing links in the chain of asthma pathogenesis. This was until epigenetics stepped into the picture. Though epigenetic research in asthma is in its nascent stages, it has led to very exciting results, especially with regard to explaining the massive influence of environment on development of asthma and its varied phenotypes. However, there remains a lot of work to be done, especially with regard to understanding how the interactions between immune system, epigenome, and environment lead to asthma. But introduction of epigenetics has infused a fresh lease of life in research into asthma and the mood among the scientific community is that of cautious optimism.

## 1. Introduction

Asthma, a chronic and recurrent disease of the airways, has over the years continued to attract the attention of the scientific community due to its widespread prevalence and associated morbidity and mortality, irrespective of age and social bearings. Even in the present day and age, the mortality figures continue to remain high [1]. Overall expenditure associated with asthma far exceeds that incurred with tuberculosis and human immunodeficiency virus (HIV) infection/acquired immunodeficiency syndrome (AIDS) [1], put together. Despite the presence of a wide variety of therapeutic options, there are none that can provide an effective cure for asthma. In light of this, the research into obtaining a better understanding of the pathophysiology and development of therapeutic options that might offer a chance at curing asthma has never let up.

Recent scientific explorations into the pathogenesis of asthma have revealed it to possess a very complex and multitiered foundation. Despite possessing a genetic component, the asthma phenotypes are not predestined or predetermined. This plasticity in asthma pathophysiology has often been held responsible for the variable phenotypes seen among asthmatics [2].

The reasons for the variability in the asthma phenotypes had often confounded the researchers. It was considered that a comprehension of the reason for variability in the asthma phenotypes could lead to a better grasp of its pathophysiology and, subsequently, newer therapeutic options. This paved the way for entry of epigenetics in asthma. However, the explorations made by the field of epigenetic research in obtaining an understanding of asthma are still in their infancy, especially in comparison to cancer. However, the mounting scientific experimental data emerging from various studies points to a growing interest in this domain [3–5].

In light of the ever burgeoning appeal of epigenetics in asthma, it is pertinent that we try to comprehend the line of thinking that indicates a possible role of epigenetics in asthma pathogenesis.

## 2. Genetics in Asthma: A False Dawn or the Stepping Stone

It had to be first ascertained that asthma had a significantly determinable genetic component in its pathophysiology. A massive study aimed at investigating the development of asthma among twins revealed that asthma development rate was about 4 times higher in monozygotic twins as compared to dizygotic twins [6]. The twin studies proved to be the ideal stepping stone for further research to be conducted and aimed at establishing a genetic angle to asthma pathophysiology. Aided by the fact that asthmatic intermediate phenotypes are highly heritable and are found to be clustered in families, extensive research into genetics in asthma was carried out. The familial inheritance of the variable asthma phenotypes was pegged at an astounding 60% [7]. The reason for the heritability has been attributed to the presence of nucleotide variants. Hence, in an effort to determine the various nucleotide variants, initially genome-wide linkage studies were carried out. These revealed a handful of genes, that is, ADAM33 [8], DPP10 [9], PHF11 [10], GPRA [11], CYFIP2 [12], HLAG [13], and PTGDR [14], to be closely associated with asthma. However, only ADAM33 and GPRA were associated with an increased incidence of development of asthma [8, 11]. Due to lack of convincing results and the limitations of genome wide linkage studies, the researchers changed course and focussed on candidate gene methods for identifying asthma associated single nucleotide polymorphisms (SNP). It is interesting to note here that this tactic yielded 300 genes containing SNPs associated with asthma [7]. The SNPs identified using candidate gene approach could lead to an increased risk in asthma development, but the actual possibility of development of asthma due to these SNPs was not found to be significant [7]. During the period of 2007–2010, about eight genome-wide association studies (GWAS) were carried out [15–21]. These GWAS have yielded information about the various new pathways that may be implicated in asthma pathophysiology and further examination could potentially throw up new candidates for drug development. Though strong associations were established between various genes identified here, the odds ratio (OR) was always within 0.5–1.5.

Despite the wealth of scientific information obtained from the linkage studies, candidate gene approach, and GWAS, most of the nucleotide variants identified till date could only be associated with a small increment in the risk of development of asthma. Additionally, careful scrutiny of the various GWAS revealed various limitations, among which neglecting the gene-environment interactions that may contribute to asthma pathogenesis has been rated as one of the major pitfalls. The results of these studies could only explain a miniscule part of the “issue” of heritability of asthma phenotypes. Hence, as a countermeasure, it was suggested that future studies should lay special focus on examining the environmental influence on the asthma phenotypes [22]. This recommendation gains significance in light of the fact that the childhood and adult onset asthma's incidence has increased substantially in the last few decades [23], which in turn suggests the possibility that gene-environment interaction may have a significant role in development of asthma. This is further substantiated by the results of the study conducted on twins [6], as mentioned earlier here. As per the study in focus here, about 19% of monozygotic twins developed asthma in concordance with one another. Now, ideally speaking, as monozygotic twins bear the same genetic constitution, they should develop asthma concordantly. However, the rate of concordance being lower than 20% in monozygotic twins is suggestive of nongenetic factors at play in the development or rather the lack of development of asthma.

## 3. Environment-Gene Interactions: The Bedrock of Epigenetics in Asthma

While the role of genetic factors in determining the susceptibility of individuals towards development of asthma is unquestionable, increasing evidence suggests a significant role of environment in shaping up of the asthmatic phenotypes. The results of a GWAS examining the effect of exposure to toluidine-diisocyanate (TDI) in Korean population go a long way in backing up this claim [17]. The results of this study, exhibiting a strong association between the gene CTNNA3 (Catenin alpha 3, alpha-T Catenin) and TDI induced asthmatic phenotype, with an OR = 5.84, hinted that the inclusion of gene-environment interactions could solve the issue of “missing heritability” in asthma. Similarly, many GWAS and preclinical and clinical studies have highlighted the potential role of environment in determination of asthma phenotypes. The first GWAS that established a strong association between asthma and 17q21 locus also revealed that there was an increased risk in the development of asthma in offspring in the families with passive exposure to environmental tobacco smoke early on in their life (OR = 2.5) as compared to families with no prior exposure to tobacco smoke (OR = 1.38). This was attributed to variants of ORMDL3 gene at rs8076131 [24, 25]. Another retrospective nested case control study suggested that prenatal exposure to tobacco smoke led to a much higher risk of development of asthma, be it childhood onset asthma or persistent asthma. The possibility of gene-environment interaction modulating the asthmatic phenotype was boosted further by an increased risk of asthma development in offspring born to maternal grandmothers with a history of smoking despite no smoking history noted in the mother's case [26].

Airway particulate matter is another major environmental factor that has been greatly studied for its impact on asthmatic phenotypes. An* in vitro* study involving human bronchial epithelial cells exhibited that exposure to diesel exhaust particulate matter could potentially bring about chromatin modifications, which in turn could produce a significant impact on the phenotype [27]. Further, analysis of a separate cohort involving elderly male subjects in the Normative Aging Study showed that level of particulate matter at work sites could possibly be correlated with various epigenetic mechanisms that in turn could modulate the respiratory phenotype of the subjects [28].

Besides environmental pollutants, dietary factors, for instance vitamin D [29], vitamin E [30], and Mediterranean diet [31], have also been examined for their effects on development of asthmatic phenotype. However, it is folic acid that has been studied most extensively for its consequences on asthma phenotypes. By supplementing folate during the period of pregnancy and weaning, airway hyperresponsiveness as well as chemokines and immunoglobulin E (IgE) production was found to be increased in animal studies [32]. Folate supplementation is strongly correlated with an increased risk of development of asthma in children [33, 34]. However, these results are in stark contrast to the results of another study that reported a positive correlation between folate intake during the gestational period and reduction in the risk of atopy and wheezing in children 2 years and older [35]. The status of folate supplementation as a major player in modulating phenotypes was further solidified when it was discovered that folate supplementation was associated with a decrease in the birth weight of newborns. The gene implicated here is insulin-like growth factor 2 (IGF-2) [36]. The recent introduction of various gut and airway microbes into the picture has complicated the interaction between various environmental factors and genes in development of asthma.

The astounding increase observed in the incidence, prevalence, and the severity of asthma in the past few decades strongly substantiates the claim that environmental exposure plays a titular role in the pathogenesis of asthma, especially via their interactions with the genetic variants. However, the rate at which the environmental exposure brings about changes can hardly be accounted for by the alterations in native DNA (deoxyribonucleic acid) sequences. An alternative explanation of this can be provided by the field of epigenetics. It deals with the study of various epigenetic marks that may be introduced into the human genome either prenatally or during various susceptible periods in the life of children, especially during newborn stage or adolescence. These epigenetic marks of the human genome can be modified more readily and rapidly by exposure to environmental factors. This can subsequently bring about modifications in the manifestations and the variability of the phenotypes in this disease. In this paper, we shed some light on the epigenetic mechanisms that could possibly vary the asthma phenotype.

## 4. Epigenetic Mechanisms in Asthma: The Means to an End

Exploration of epigenetics in the field of asthma is in its nascent stages. The paucity of studies at this stage has led to the epigenetic epidemiological data about asthma trickling in slowly. The question that arises now is how epigenetic mechanisms bring about various alterations in the asthma phenotype leading to the wide variability in this disease.

The possible epigenetic mechanisms could involve DNA methylation, posttranslational histone alteration, and noncoding RNA (ribonucleic acid) dysregulation. However, the most commonly implicated mechanism in most of the studies has been DNA methylation induced genetic dysregulation. There are various theories that have been propounded in a bid to explain the potential role of DNA methylation in the development of asthma. Among them, the commonly explored one is when exposure to particulate matter leads to demethylation or rather hypomethylated state of long interspersed nucleotide elements (LINE-1) [28, 37] and* Arthrobacter luteus *(Alu) repeated elements [37], which subsequently leads to the activation of various promoter genes in these genetic segments and increased incidences of genomic alterations and instability and transcriptional dysregulation [38, 39]. It has been hypothesised that exposure to air particulate matter through catalytic redox cycling may increase the production of reactive oxygen species (ROS) [40]. The oxidative damage produced by these ROS prevents the interaction between DNA and methyltransferases enzyme, leading to hypomethylated CpG sites [41]. Besides altering the interaction between DNA and methyltransferases enzymes, metal exposure could induce crucial alterations in the DNA methylation machinery itself [42]. One group of researchers observed that cadmium could inhibit the activity of DNA methyltransferases by attaching to the methyltransferases binding site on DNA and subsequently interfering with the DNA and methyltransferases interaction [43]. Cellular stores of methyl groups tend to undergo depletion on exposure to arsenic, possibly aiding in the hypomethylation of DNA [42]. The hypomethylation of various repetitive elements and subsequent transcriptional dysregulation has been shown to be associated with cellular stress [44] and alveolar inflammation [40].

In case of asthma specific candidate gene for inducible nitric oxide synthase (iNOS), exposure to particulate matter [37] could lead to demethylation of iNOS gene. Consequently, this may lead to increased expression of proinflammatory iNOS leading to respiratory and cardiovascular inflammatory states. Though any study is yet to shed any light on the mechanism by which particulate matter can demethylate iNOS gene, there is a high probability that it might be mediated via the ROS action as suggested in a study [45].

A first of a kind proof of concept study conducted by the Columbia Center for Children's Environmental Health (CCCEH) was able to draw out a correlation between exposure to polycyclic aromatic hydrocarbons (PAHs) and methylation of acyl-CoA synthetase long chain family member 3 (ACSL3) gene [46]. ACSL3 gene, associated with fatty acid metabolism, primarily expressed in lung and thymus, was identified as an original potential epigenetic marker for environmentally induced asthmatic state. Acyl-CoA synthetase is essential for production of acyl-CoA that can be used for both of intracellular lipids and, simultaneously, their degradation through beta oxidation yielding energy [47, 48]. Additionally, acyl-CoA synthetase is critical to phospholipid modification in the burgeoning T cells [49]. It has been hypothesised that hypermethylation of this singular gene could potentially have far-reaching effects in the pathophysiology of asthma. Prenatal exposure of PAH was associated with increased methylation status of ACSL3 gene, which in turn has been associated with increased incidences of development of childhood asthma [46]. The question about how various functional alterations brought about by hypermethylation of this gene impact asthma remains unanswered. Further mechanistic studies are needed to delineate out the role of ACSL3 in the development of asthma, especially childhood asthma. However, the recent reports on ACSL3 as a potential epigenetic marker for prediction of PAH associated asthma provide the first yet important step in this research domain.

Development of asthma also involves a key transcription factor modulating the activity of T regulatory cells; that is, Forkhead box transcription factor 3 (Foxp3). T regulatory cells are involved in the initial steps of sensitization to allergens and IgE production consequent to exposure to allergens [50]. It has been suggested that exposure to air pollutants may bring about methylation of promoter regions involving the Foxp3 gene, which decreases the expression of Foxp3 and subsequently the development and functioning of T regulatory cells [51]. Expression of chemokine receptors CCR4 and CCR8 on T regulatory cells is governed by Foxp3 as well [52]. These chemokine receptors may be critical for guiding the movement of T regulatory cells to the bronchial epithelium [53]. This is substantiated by the reduced number of T regulatory cells observed in the bronchoalveolar lavage fluid of asthmatic subjects [54], reduced number of circulating T regulatory cells [55], and impairment in chemotaxis of T regulatory cells to respiratory epithelial cells [56]. Consequently, Foxp3 methylation hints towards worsening of asthma pathology due to impaired production and functioning of T regulatory cells, thus providing a very interesting mark that can be targeted while exploring potential epigenetic therapeutic options against asthma.

There is a heap of evidence to suggest that the adaptive immune programming seen in the pathogenesis of asthma may be amenable to epigenetic modifications [57–61]. It has been observed that, at its resting state, the IL-4, that is, T helper 1 (Th1) cytokine, and IFN-*γ*, that is, T helper 2 (Th2) cytokine genes in a naïve CD4 T cell, are methylated [62]. However, exposure to an allergen can tilt the balance between Th1 and Th2 responses in favour of proallergic Th2 cytokine responses by demethylating the IL-4 promoter [62]. The demethylation of IL-4 locus is strongly associated with the IL-4 cytokine expression, subsequently leading to STAT6 phosphorylation resulting in the activation of the master regulatory GATA-3 gene and, finally, increasing the expression of IL-4 [63]. Further, GATA-3 activation has also been shown to repress TBET expression, which is the Th1 differentiation master regulator. GATA-3 has been shown to increase the methylation status of IFN-*γ* locus as compared to their naïve state [64]. Thus, DNA hypomethylation of the CpG promoter sites of IFN-*γ* could activate the expression of IFN-*γ*. This counterregulatory cytokine could provide a protective cover against the proallergic cytokines [61]. However, insights into how the DNA methylation or hypomethylation of the IL-4 and IFN-*γ* promoter sites is carried out are lacking.

Besides DNA methylation, studies on histone modifications have also revealed interesting marks that could serve as potential therapeutic targets. Experimentation with HDAC inhibitors, for instance, Trichostatin A, has been associated with Th2 skewing and enhanced GATA-3 expression, which is suggestive of the crucial role that HDAC may play in maintaining the Th1 versus Th2 balance [65]. The deliberations over the potential protective role of HDAC against proallergic Th2 cytokine production received a shot in the arm when it was observed by the same group of researchers that histone acetyl transferase (HAT) activity was significantly elevated and HDAC expression was reduced in atopic asthmatics as compared to atopic nonasthmatics. The levels of HAT and HDAC were correlated strongly with bronchial hyperreactivity in this study, thus establishing pioneering work in drawing an association between epigenetic modifications and a phenotype that can be observed clinically and measured [65]. However, the role of HAT and HDAC in the pathogenesis of asthma is not so straightforward. An interesting study exploring the protection provided by the Gram-negative bacterium* Acinetobacter lwoffii* F78 has attributed it to increased H4 acetylation of the IFN-*γ* promoter site, which was found to be missing on treatment with a HAT inhibitor [66]. Another experiment demonstrated a strong correlation between T regulatory cell induction and HDAC inhibition by various bacterial metabolites which in turn was associated with decreased proinflammatory cytokine expression in dendritic cells [67]. But in cumulation, via the results emerging from these studies and many others in this domain of epigenetic research, it has been established beyond reproach that both DNA methylation and histone modifications are two extremely influential and flexible tenets of determination of T helper cell lineages, which may find application in prevention of asthma.

Finally, our discourse on the epigenetic mechanisms that have been speculated to contribute to asthma pathogenesis would remain incomplete without a mention of the microRNAs (miRNAs). Though not as extensively researched compared to DNA methylation and histone modification, recent animal and even human studies have revealed a potentially significant role of miRNAs in asthma pathogenesis. It has been suggested that the robust nature and tissue specific distribution of miRNAs could find utility in identification of “at-risk” individuals for asthma [68]. As reported in a few murine studies, miRNA profiling here has helped identify a few potential biomarkers in asthma pathogenesis [69, 70]. In addition to these animal studies, abnormal profiles of miRNA have been examined in humans too. A study showed that there were dramatic alterations in the expression patterns of 200 and above miRNAs among asthmatics [71]. A large majority of these remain uncorrected or modestly corrected despite treatment with steroids [71]. Another recent study revealed that about 26 miRNAs are aberrantly expressed in the bronchial smooth muscle cells of asthmatic cells [72]. Besides, the target mRNAs of these aberrantly regulated miRNAs were found to play important roles in asthma pathogenesis in the form of cellular proliferation through phosphatase and tensin homolog and phosphoinositide 3-kinase/Akt signaling pathways. Thus, these mRNAs which were being looked at as potential antiasthma targets have now led us to multiple miRNAs that could be manipulated to control asthma [72]. These study results are not in isolation. Similarly, evidences have also emerged from various other studies that have shed light on the potential therapeutic role of miRNAs in asthma. An animal study pointed to a role of miR-223 in granulocyte production and inflammatory response [73]. Further, as per another murine study, another miRNA, miR-126's blockade, was found to be associated with diminished Th2 cellular responses in the lung, leading to decreased inflammation, eosinophilic recruitment, and hypersecretion of mucus [74]. An elegant* in silico* study revealed that targeting miR-9 could potentially find utility in treatment of steroid resistant asthma [75]. The authors have hypothesised that miR-9 through its role in the glucocorticoid signaling pathway could serve as a novel target for treatment of steroid resistant asthma [75]. These encouraging evidences from the preliminary studies point to a potential diagnostic, prognostic, and therapeutic role of miRNAs in asthma.

## 5. Perspectives and Conclusion

Despite the many theories like the hygiene hypothesis and triggers like allergens, diet, and so forth that have been identified to play a significant role in the development of asthma, none of them come close to providing a unified well-developed mechanism that can account for the majority of these triggers. Epigenetic mechanisms not only have opened up a new and a different field for exploration in asthma but also strive to explain most of the existing theories concerned with asthma. Further, reversibility of potential epigenetic therapies lends an added advantage to it. Epigenetics also attempts to understand the complex gene-environment interactions that have often confounded the researchers for so long. Utilising epigenetic principles, various critical marks that are linked closely to asthma and interact with environmental factors can be identified and can serve as the template for development of potential therapeutic options. In addition, obtaining an understanding of the complex epigenetic-environment interactions can aid in formulating interventions for at-risk individuals and help prevent the development of asthma in the first place.

However, the complexity of asthma as a disease poses a significant challenge towards filling in the missing pieces of the puzzle of asthma pathogenesis. Though the contribution of epigenetics in clarifying the earlier confounding aspects of asthma pathogenesis has been significant, the real challenge that lies ahead is in understanding how the genetic variability, epigenetic marks, environment, transcriptome, and the adaptive immune system interact with one another to produce different types of asthma phenotypes. Besides this, there too remains significant work to be done in understanding the effects of epigenetic regulatory mechanisms in different cells. There is also the additional aspect of limitations of the designs of various studies that have been conducted till date. The cohorts examined in various studies have been small and have not been sufficient to extrapolate the results to clinical settings. However, epigenetics in asthma promises potentially exciting rewards and it is hoped that the research work that has been limited to only the laboratories until now can soon take a leap forward and find application in the clinical settings.
